# Correlation analysis of serum FGF9, Sestrin2 and HBDH with the severity of sepsis

**DOI:** 10.5937/jomb0-61187

**Published:** 2026-05-22

**Authors:** Jian He, Xinru Lin, Xiang Ding, Maoxia Liu

**Affiliations:** 1 Taikang Xianlin Drum Tower Hospital, Affiliated Hospital of Medical School, Nanjing University, Department of Emergency Medicine, Nanjing, China; 2 Taikang Xianlin Drum Tower Hospital Clinical College of Wuhan University, Department of Emergency Medicine, Nanjing, China; 3 The Second Affiliated Hospital of Anhui Medical University, Department of Infectious Diseases, Economic and Technological Development Zone, Hefei City, China; 4 Chongqing University Central Hospital (Chongqing Emergency Medical Centre), Chongqing, China

**Keywords:** sepsis, fibroblast growth factor 9, Sestrin2, hydroxybutyrate dehydrogenase, correlation analysis, sepsa, faktor rasta fibroblasta 9, Sestrin2, hidroksibutirat dehidrogenaza, analiza korelacije

## Abstract

**Background:**

To explore the correlation between serum fibroblast growth factor 9(FGF9), Sestrin2 and hydroxybutyrate dehydrogenase (HBDH) and the severity of sepsis, and to analyse the predictive value of FGF9, Sestrin2 and HBDH for clinical outcome.

**Methods:**

A retrospective analysis of 125 patients with sepsis admitted to Taikang Xianlin Drum Tower Hospital and Chongqing University Central Hospital between May 2022 and May 2025 was conducted. According to disease severity, there were 50 cases in the septic shock group and 75 in the sepsis group. Based on clinical outcomes, patients were divided into a death group (103 cases) and a survival group (22 cases). Observe the levels of serum FGF9, Sestrin2 and HBDH in each group. Analysis of the relationships among FGF9, Sestrin2, HBDH, and sepsis severity was performed using the Spearman correlation coefficient. Using multivariate logistic regression analysis, the factors influencing the prognosis of patients with sepsis were investigated. FGF9, Sestrin2, and HBDH were evaluated for their prognostic utility in sepsis patients using receiver operating characteristic (ROC) curves.

**Results:**

Compared with the sepsis group, the level of FGF9 in the septic shock group was lower [(122.41± 21.45) pg/mL vs (145.36± 23.68) pg/mL], while the levels of Sestrin2 and HBDH were both higher [(15.75± 2.34) ng/mL] vs (8.36± 0.93) ng/mL, (232.14± 34.77) U/L vs (166.25± 24.85) U/L]; the differences were statistically significant (P&lt; 0.05). Compared with the survival group, the level of FGF9 in the death group was lower [(112.05± 20.61) pg/mL vs (133.25± 22.14) pg/mL], while the levels of Sestrin2 and HBDH were both higher [(19.25± 2.85) ng/mL] vs (12.42± 2.33) ng/mL, (261.25± 37.25) U/L vs (204.25± 32.18) U/L]. Spearman correlation coefficient analysis showed that FGF9 was negatively correlated with the severity of sepsis (P&lt; 0.05), whereas the degree of sepsis was positively correlated with both Sestrin2 and HBDH (P&lt; 0.05). Sequential Organ Failure Assessment (SOFA), Acute Physiology and Chronic Health Evaluation II (APACHE II), procalcitonin, FGF9, Sestrin2, and HBDH are risk factors for sepsis prognosis, according to multivariate logistic regression analysis (P&lt; 0.05). The results of ROC curve analysis showed that the combined detection of FGF9, Sestrin2 and HBDH had a better predictive effect on the prognosis of patients with sepsis (P&lt; 0.05).

**Conclusions:**

The amount of FGF9 in septic shock patients is comparatively low, while the levels of Sestrin2 and HBDH are relatively high. As the severity of the disease increases, the level of FGF9 gradually decreases, while the levels of Sestrin2 and HBDH gradually increase, with a certain correlation with disease severity. The prognosis of sepsis patients can be assessed by measuring FGF9, Sestrin2, and HBDH levels.

## Abstract (sr)

**Uvod:**

Cilj je bio da se istraži korelacija između serumskog faktora rasta fibroblasta 9 (FGF9), Sestrina2 i hidroksibutirat dehidrogenaze (HBDH) sa težinom sepse, i analizira prediktivna vrednost FGF9, Sestrina2 i HBDH u odnosu na klinički ishod.

**Metode:**

Sprovedena je retrospektivna analiza koja je uključila 125 pacijenata sa sepsom primljenih u Taikang Xianlin Drum Tower bolnicu i Centralnu bolnicu Univerziteta u Chongqingu u periodu od maja 2022. do maja 2025. godine. Prema težini bolesti, bilo je 50 slučajeva u grupi sa septičkim šokom i 75 u grupi sa sepsom. Na osnovu kliničkih ishoda, pacijenti su podeljeni u grupu sa smrtnim ishodom (103 slučaja) i grupu preživelih (22 slučaja). Posmatrani su nivoi serumskog FGF9, Sestrina2 i HBDH u svakoj grupi. Analiza odnosa između FGF9, Sestrina2, HBDH i težine sepse sprovedena je korišćenjem Spirmanovog koeficijenta korelacije. Faktori koji utiču na prognozu pacijenata sa sepsom ispitani su multivarijatnom logističkom regresijom. Prognostička vrednost FGF9, Sestrina2 i HBDH procenjivana je pomoću ROC krivih.

**Rezultati:**

U poređenju sa grupom sa sepsom, nivo FGF9 u grupi sa septičkim šokom bio je niži [(122,41± 21,45) pg/mL vs (145,36± 23,68) pg/mL], dok su nivoi Sestrina2 i HBDH bili viši [(15,75± 2,34) ng/mL vs (8,36± 0,93) ng/mL, (232,14± 34,77) U/L vs (166,25± 24,85) U/L]; razlike su bile statistički značajne (P&lt; 0,05). U poređenju sa grupom preživelih, nivo FGF9 u grupi sa smrtnim ishodom j'e bio niži [(112,05± 20,61) pg/mL vs (133,25± 22,14) pg/mL], dok su nivoi Sestrina2 i HBDH bili viši [(19,25± 2,85) ng/mL vs (12,42± 2,33) ng/mL, (261,25± 37,25) U/L vs (204,25± 32,18) U/L]. Analiza Spearmanovog koeficijenta korelacije pokazala je da je FGF9 negativno korelisao sa težinom sepse (P&lt; 0,05), dok je stepen sepse bio pozitivno korelisan sa Sestrinom2 i HBDH (P&lt; 0,05). Multivarijatna logistička regresija pokazala je da SOFA (Sequential Organ Failure Assessment), APACHE II (Acute Physiology and Chronic Health Evaluation II), prokalcitonin, FGF9, Sestrin2 i HBDH predstavljaju faktore rizika za prognozu sepse (P&lt; 0,05). Rezultati ROC analize su pokazali da kombinovano određivanje FGF9, Sestrina2 i HBDH ima bolju prediktivnu vrednost za prognozu pacijenata sa sepsom (P&lt; 0,05).

**Zaključak:**

Količina FGF9 kod pacijenata sa septičkim šokom je relativno niska, dok su nivoi Sestrina2 i HBDH relativno visoki. Kako težina bolesti raste, nivo FGF9 postepeno opada, dok nivoi Sestrina2 i HBDH postepeno rastu, pri čemu postoji određena korelacija sa težinom bolesti. Prognoza u slučaju pacijenata sa sepsom se može odrediti merenjem nivoa FGF9, Sestrina2 i HBDH.

## Introduction

A systemic inflammatory reaction condition brought on by an infection called sepsis, with a relatively high clinical mortality rate, which can pose a significant threat to patients' lives and safety [1][2][3]. With the increase in the number of clinically obese and diabetic patients, the drug resistance of pathogenic bacteria is gradually rising, which can affect the treatment effect of sepsis patients [4]. Consequently, patients' prognoses can be improved and the clinical treatment rate raised by early intervention to avert septic shock. Fibroblast growth factor-9 (FGF9) is an inflammation-related growth factor that can regulate various inflammatory diseases, inhibit the occurrence of sepsis, and reduce inflammatory infiltration of the alveolar wall. The stress-induced protein Sestrin2 can regulate inflammatory and stress responses within the body, causing autophagy in cells [5][6][7]. An increase in Sestrin2 levels can promote the development of septic shock. Hydroxybutyrate dehydrogenase (HBDH) is mainly distributed in myocardial cells [8][9][10]. After myocardial cells are damaged, the level of HBDH can increase, leading to myocardial injury in patients with sepsis and increasing the incidence of clinical death.

Sepsis is a systemic inflammatory response to infection. In clinical practice, it is a prevalent catastrophic illness, with a high incidence and mortality rate [11][12][13]. Early identification of sepsis severity and timely adoption of effective treatment measures are crucial for improving patient prognosis. However, the commonly used sepsis assessment indicators in clinical practice at present, such as the SOFA and APACHE II scores, although they have some predictive value, still have limitations, including significant subjectivity and long-time consumption. Therefore, the search for new, objective biomarkers that reflect the severity of sepsis at an early stage is of substantial clinical importance [14]. In recent years, the roles of fibroblast growth factor 9 (FGF9), Sestrin2, and 3-hydroxybutyrate dehydrogenase (HBDH) in various diseases have attracted increasing attention. FGF9 is a multifunctional growth factor that participates in processes such as cell proliferation, differentiation and migration. Studies [15][16][17] have shown that it plays a vital role in inflammatory responses. Sestrin2 is a stress-induced protein with functions including antioxidation, anti-apoptosis, and metabolic regulation. HBDH is a key enzyme in ketone body metabolism, and changes in its activity may reflect disturbances in energy metabolism in patients with sepsis. Preliminary research [18] suggests that the incidence and progression of sepsis may be correlated with levels of FGF9, Sestrin2, and HBDH, but the relationship between these factors and sepsis severity remains unclear.

This study detected and analysed serum levels of FGF9, Sestrin2, and HBDH in patients with sepsis and explored their relationships with disease severity and clinical outcomes.

## Materials and methods

### General information and grouping

A retrospective analysis of 125 sepsis patients admitted to Taikang Xianlin Drum Tower Hospital and Chongqing University Central Hospital between May 2022 and May 2025 was conducted. The patients were divided into 75 cases in the sepsis group and 50 in the septic shock group based on illness severity, in accordance with the third definition of sepsis and septic shock, which is the international consensus standard. Based on clinical outcomes, patients were divided into a death group (103 cases) and a survival group (22 cases). The general patient data across groups were comparable and did not differ significantly (P>0.05).

### Inclusion and exclusion criteria

Inclusion criteria: (1) It complies with the diagnostic criteria stipulated in the »Expert Consensus on Early Prevention and Blockade of Sepsis in Emergency Departments of China«; (2) Complete clinical data; (3) Clear consciousness and mind, with normal language communication skills.

Exclusion criteria: (1) Death within 24 hours of hospitalisation; (2) Long-term use of immunosuppressants for treatment; (3) Suffering from malignant tumours; (4) Combined with end-stage renal disease; (5) Combined with haematological and autoimmune diseases; (6) During pregnancy or lactation.

### Detection of serum FGF9, Sestrin2 and HBDH levels

Five mL of the patient's fasting elbow venous blood was collected in the morning. The operation was carried out at 4°C using a centrifuge (15 min, 3,500 r/min, 5 cm radius). The supernatant was separated and stored at -80°C for future use. Serum levels of FGF9, Sestrin2, and HBDH were measured by enzyme-linked immunosorbent assay. Sample Wells, standard Wells and blank Wells were designed in the microplate. The sample Wells were filled with 50 μL of the test sample, and then 50 μL of FGF9, Sestrin2 and HBDH standards of different concentrations were added to the standard Wells. Add 50 μL of HRP to each well and incubate at room temperature for 1 hour. Then add washing buffer and wash 5 times. Incubate each well for 15 minutes with 50 μL of substrates A and B at 37°C. With an enzyme-linked immunosorbent assay (ELISA) reader, add the stop solution and measure the standard Wells' absorbance value at 450 nm. HBDH, FGF9, and Sestrin2 levels were found.

### Data collection

### Conduct statistics on patient data

General information: Age, gender, body mass index, diabetes, hypertension, smoking, site of infection (urinary system, lungs, others).

### Biochemical indicators

Acute Physiology and Chronic Health Evaluation II (APACHE II) score, serum creatinine, procalcitonin, white blood cell count, neutrophil count, platelet count, and sequential organ failure assessment (SOFA) score.

### Observation indicators

The levels of serum FGF9, Sestrin2 and HBDH in patients of the sepsis group, septic shock group, survival group and death group. Analyse the correlation between serum FGF9, Sestrin2, HBDH and the severity of sepsis. Analyse the elements that affect sepsis patients' prognosis and their prognostic value for such patients' outcomes.

### Statistical analysis

Data from the study were analysed using SPSS 20.0. The mean ± standard deviation (x̄±s) was used for the measurement data. Analysis of variance was used to compare results across many groups, whereas the t-test was used to compare two groups. While analysis of variance was employed for comparisons among many groups, the t-test was used for comparisons between two groups. The χ^2^ test was used to compare groups, and counts were presented as cases or percentages (%). The correlation was examined using the Spearman correlation coefficient model. Factors affecting the prognosis of sepsis patients were examined using multivariate logistic regression. Sensitivity, specificity, confidence intervals, and the area under the receiver operating characteristic (ROC) curve were used to assess the predictive value of FGF9, Sestrin2, and HBDH for sepsis prognosis.

## Results

### Serum levels of FGF9, Sestrin2, and HBDH were compared between the two patient groups

The serum FGF9 level of patients in the septic shock group was significantly lower than that in the sepsis group, while the levels of Sestrin2 and HBDH were significantly higher than those in the sepsis group, suggesting that during the progression of sepsis to septic shock, the FGF9 level decreased, while the levels of Sestrin2 and HBDH increased. The death group's serum FGF9 levels were likewise noticeably lower than those of the survival group, according to an additional study, while the levels of Sestrin2 and HBDH were significantly higher than those in the survival group, indicating that the levels of FGF9, Sestrin2 and HBDH are closely related to the prognosis of patients with sepsis.

The further reduction of FGF9 and the further increase of Sestrin2 and HBDH in patients of septic shock and death group may indicate disease deterioration and poor prognosis. Therefore, the differences in serum FGF9, Sestrin2 and HBDH levels may reflect the severity of the condition and prognostic risk in patients with sepsis.

The septic shock group had lower FGF9 levels than the sepsis group, while Sestrin2 and HBDH levels were higher (Table 1).

**Table 1:** Serum FGF9, Sestrin2, and HBDH levels between two groups of sepsis patients (x̄ ± s).

Group	Number of cases	FGF9 (pg/mL)	Sestrin2 (ng/mL)	HBDH (U/L)
Sepsis group	75	145.36±23.68	8.36±0.93	166.25±24.85
Septic shock group	50	22.41±21.45	15.75±2.34	232.14±34.77
t value		5.509	24.626	12.356
P value		<0.001	<0.001	<0.001

### Serum FGF9, Sestrin2 and HBDH levels in patients with different prognoses

Patients in the death group had lower levels of FGF9 than those in the survival group, while the Sestrin2 and HBDH levels were higher. According to Table 2, the differences were statistically significant (P<0.05). Patients in the death group had much lower serum FGF9 levels than those in the survival group, although they had significantly greater levels of Sestrin2 and HBDH. This finding suggests that serum levels of FGF9, Sestrin2, and HBDH are closely associated with the prognosis of patients with sepsis. Lower FGF9 levels and higher Sestrin2 and HBDH levels may indicate a poor prognosis for patients and an increased risk of death.

**Table 2:** Serum FGF9, Sestrin2, and HBDH levels in patients with sepsis with different prognoses (x̄ ± s).

Group	Number of cases	FGF9 (pg/mL)	Sestrin2 (ng/mL)	HBDH (U/L)
Survival group	22	133.25±22.14	12.42±2.33	204.25±32.18
Death group	103	112.05±20.61	19.25±2.85	261.25±37.25
t value		4.323	10.505	6.661
P value		0.001	<0.001	<0.001

### The correlation between serum FGF9, Sestrin2 and HBDH and the severity of sepsis

There is a strong correlation between sepsis severity and serum levels of FGF9, Sestrin2, and HBDH. The degree of sepsis is inversely correlated with FGF9 levels: as sepsis worsens, FGF9 levels gradually decrease. A comparison between the sepsis and septic shock groups further supported this pattern. Compared with the sepsis group, the FGF9 level in the septic shock group was noticeably lower, while the Sestrin2 and HBDH levels were significantly higher than those in the sepsis group.

The severity of the disease was taken as a categorical variable. A correlation analysis was conducted with sepsis as 1 and septic shock as 2. FGF9 was negatively correlated with the severity of sepsis (P<0.05), while Sestrin2 and HBDH were positively correlated with the severity of sepsis (P<0.05) (Table 3).

**Table 3:** Correlation between serum FGF9, Sestrin2, HBDH and the severity of sepsis.

Indicator	Severity	
	r value	P value
FGF9	-0.851	<0.001
Sestrin2	0.625	0.004
HBDH	0.733	<0.001

### Univariate investigation of the variables affecting sepsis patients' prognosis

Regarding gender, age, body mass index, smoking, diabetes, hypertension, or infection site, there were no statistically significant differences between the two groups (P>0.05). Compared with the survival group, the SOFA score, APACHE II score, procalcitonin, Serum creatinine, neutrophil count, and white blood cell count were all greater in the death group, while the platelet count was lower (Table 4).

**Table 4:** Factors influencing the prognosis of sepsis patients with univariate analysis.

Data	Survival group<br>(n = 22)	Death group<br>(n = 103)	t/F/x^2^ value	P value
Gender [Example (%)]			0.292	0.589
Male	11 (50.00)	58 (56.31)		
Female	11 (50.00)	45 (43.69)		
Age (Years, x̄±s)	65.25±3.58	65.31±3.60	0.071	0.944
Body mass index (kg/m^2^, x̄±s)	22.25±4.11	22.21±4.08	0.042	0.967
Smoking [Cases (%)]			1.231	0.267
Yes	12 (54.55)	69 (66.99)		
No	10 (45.45)	34 (33.01)		
Diabetes [Cases (%)]			2.894	0.089
Yes	8 (36.36)	58 (56.31)		
No	14 (63.64)	45 (43.69)		
Hypertension [Cases (%)]			0.458	0.498
Yes	10 (45.45)	55 (53.40)		
No	12 (54.55)	48 (46.60)		
Infection site [Cases (%)]			0.303	0.762
Urinary system	8 (36.36)	42 (40.78)		
Lung	7 (31.82)	30 (29.12)		
Others	7 (31.82)	31 (30.10)		
SOFA score (Points, x̄±s)	5.36±0.64	11.25±2.48	1.029	<0.001
APACHE II score (Points, x̄±s)	13.12±2.44	19.25±2.85	9.374	<0.001
Procalcitonin (ng/mL, x̄±s)	2.36±0.34	12.52±3.44	13.795	<0.001
White blood cell count (×10^9^/L, x̄±s)	8.12±0.93	14.12±2.38	11.606	<0.001
Neutrophil count (×10^9^/L, x̄±s)	70.36±8.33	88.25±9.27	8.355	<0.001
Platelet count (×10^9^/L, x̄±s)	194.36±28.41	142.23±23.22	9.177	<0.001
Serum creatinine (μmol/L, x̄±s)	85.14±9.23	91.25±10.11	2.611	0.010

### Multivariate logistic regression analysis was used to look at the factors influencing the prognosis of sepsis patients

The prognosis of sepsis patients was considered the dependent variable, and factors with P<0.05 in the univariate analysis were selected as independent variables for assignment (survival = 0; death = 1). A logistic regression analysis with many variables was then performed. According to multivariate results, serum creatinine, procalcitonin, white blood cell, neutrophil, and platelet counts, FGF9, Sestrin2, HBDH, APACHE II score, SOFA score, and APACHE score are risk variables influencing the prognosis of sepsis patients (P<0.05) (Table 5).

**Table 5:** Multivariate logistic regression analysis of factors influencing the prognosis of patients with sepsis.

Factor	β value	SE value	Wald χ^2^ value	OR value	95% CI	P value
SOFA score	1.859	0.621	8.961	6.417	5.175~7.659	0.005
APACHE II score	1.215	0.314	15.873	3.494	2.866~4.122	<0.001
Procalcitonin	1.452	0.485	8.963	4.272	3.302~5.242	0.004
White blood cell count	1.332	0.214	38.742	3.7893	.361~4.217	<0.001
Neutrophil count	1.251	0.332	14.198	3.494	2.830~4.158	<0.001
Platelet count	0.332	0.125	7.054	1.394	1.144~1.644	0.008
Serum creatinine	1.458	0.221	43.524	4.297	1.926~2.782	<0.001
FGF9	0.856	0.214	16.000	2.354	1.926~2.782	<0.001
Sestrin2	1.552	0.321	23.376	4.721	4.079~5.363	<0.001
HBDH	1.052	0.336	9.803	2.863	2.191~3.535	<0.001

### The prognostic value of FGF9, Sestrin2, and HBDH for the prognosis of sepsis patients was examined using a ROC curve

**Figure 1:**
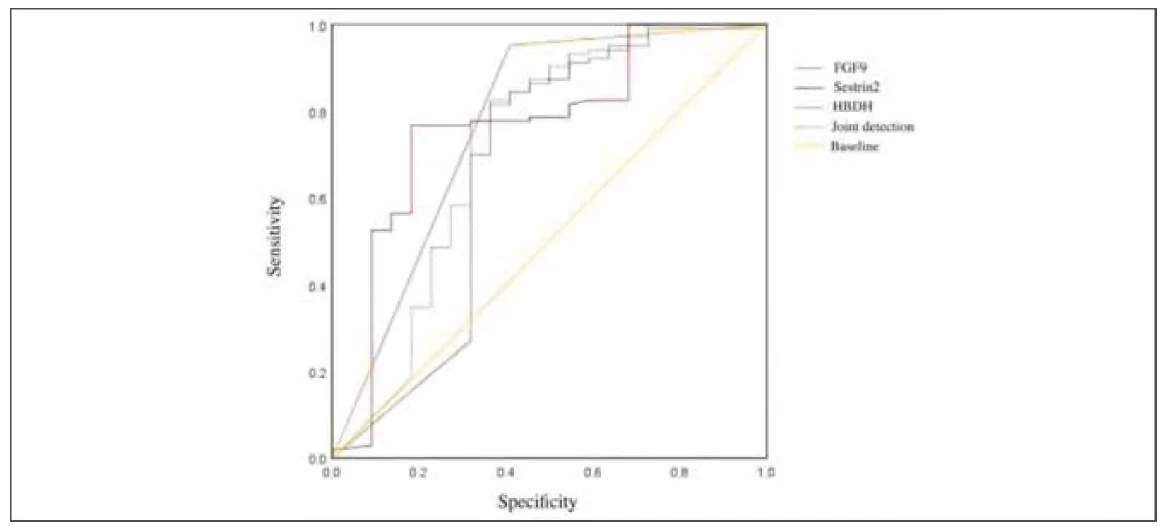
ROC curve analysis was used to predict the prognosis of patients with sepsis by FGF9, Sestrin2 and HBDH.

The area under the curve of the effect of individual detection of FGF9, Sestrin2 and HBDH in predicting the prognosis of patients with sepsis. The AUC values were 0.718, 0.764, and 0.669, respectively. When FGF9, Sestrin2, and HBDH were detected together, the AUC was 0.771, all of which were higher than those of the individual detections of FGF9, Sestrin2, and HBDH, and had good predictive value (P<0.05) (Table 6 and Figure 1).

**Table 6:** ROC curve analysis of the predictive value of FGF9, Sestrin2, and HBDH for the prognosis of patients with sepsis.

Project	AUC sensitivity (%)	Specificity (%)	Accuracy (%)	95%CI	P value
FGF9	0.71879.61 (82/103)	68.18 (15/22)	75.20 (94/125)	0.572~0.864	0.001
Sestrin2	0.7647.67 (80/103)	81.82 (18/22)	78.40 (98/125)	0.643~0.885	0.001
HBDH	0.66982.52 (85/103)	63.64 (14/22)	79.20 (99/125)	0.510~0.829	0.002
Joint testing	0.77195.15 (98/103)	59.09 (13/22)	88.80 (111/125)	0.641~0.902	0.001

## Discussion

A systemic inflammatory illness known as sepsis develops when the body becomes infected with harmful bacteria [19][20][21]. It can affect multiple organs. As the disease progresses, septic shock may occur, causing a drop in blood pressure in patients, damaging lung, liver and kidney functions, and threatening the patient's life. The clinical assessment of sepsis prognosis primarily uses the SOFA and APACHE II scores [22]. However, this scoring process is complex and easily influenced by subjective factors, thereby reducing prediction accuracy. Consequently, it is essential to look for signs associated with sepsis, assess its development and prognosis accurately, determine the severity of the disease, predict the clinical prognosis, and carry out timely treatment interventions [23][24][25].

FGF9 is a polypeptide growth factor with a wide range of biological functions in clinical settings. It can participate in various biological processes, promote cell development, and facilitate tissue repair. When its expression level is abnormal, it can contribute to the development of many diseases. FGF9 can promote lipid accumulation in cells and regulate liver lipid metabolism through autocrine signalling. Moreover, FGF9 expression can affect the body's inflammatory response [26].

After FGF9 activates the phosphatidylinositol-3-kinase serine protein kinase pathway, it can alleviate the inflammatory response and inhibit the development of diseases. In patients with septic shock, FGF9 expression is relatively low. The level of FGF9 gradually decreases as disease severity increases [27][28][29]. Sestrin2 is an oxidative stress-inducing protein and a key molecule in the antioxidant system. Moreover, Sestrin2 can promote the phosphorylation of adenosine monophosphate-activated protein kinase, increase hydrogen peroxide levels, inhibit reactive oxygen species expression, and reduce oxidative stress-induced cell damage [30]. After the body undergoes hypoxia and oxidative stress, Sestrin2 expression can increase, exerting a protective effect. Relevant studies have shown that in patients with sepsis, Sestrin2 has a protective effect, inhibiting its expression and increasing sepsis mortality. The possible reason is that Sestrin2 levels increase in septic shock, which may be driven by an excessive inflammatory response and increased oxidative stress, and this is an endogenous protective effect [31].

HBDH is widely distributed throughout the body. After septic shock occurs, it can alter hemodynamics and cause microcirculatory disorders, leading to myocardial ischemia, diffuse swelling, and myocardial cell disintegration and damage. Clinical studies [32][33][34] have shown that after myocardial cell injury, the contractile protein myosin can be involved, reducing myocardial contractility, causing myocardial cell disintegration, and promoting the release of HBDH. Moreover, abnormal HBDH expression can affect myocardial cells in patients, leading to myocardial ischemia and injury and a poor prognosis.

The ROC curve shows that the combined detection of FGF9, Sestrin2, and HBDH has a sensitivity of 95.15%, a specificity of 59.09%, and an accuracy of 88.80% in predicting the prognosis of patients with sepsis, indicating high predictive value. It suggests that detecting FGF9, Sestrin2, and HBDH can predict clinical outcomes in patients with sepsis and has a relatively high practical value.

## Conclusion

In patients with sepsis, FGF9 levels are relatively low, while Sestrin2 and HBDH levels are relatively high. As disease severity worsens, FGF9 levels decrease, while Sestrin2 and HBDH levels increase, suggesting a correlation with disease severity. Moreover, FGF9, Sestrin2 and HBDH are risk factors leading to the death of patients. Clinically, monitoring FGF9, Sestrin2, and HBDH can predict the prognosis of patients with sepsis and support early diagnosis and treatment, with substantial clinical value.

## Dodatak

### Funding

This work is supported by »Clinical study on the early prediction of community-acquired sepsis by H LA-DR combined with cytokines« [TKKYZX20241513].

### Conflict of interest statement

All the authors declare that they have no conflict of interest in this work.
